# Two Legendre-Dual-Petrov-Galerkin Algorithms for Solving the Integrated Forms of High Odd-Order Boundary Value Problems

**DOI:** 10.1155/2014/309264

**Published:** 2014-01-23

**Authors:** Waleed M. Abd-Elhameed, Eid H. Doha, Mahmoud A. Bassuony

**Affiliations:** ^1^Department of Mathematics, Faculty of Science, King Abdulaziz University, Jeddah, Saudi Arabia; ^2^Department of Mathematics, Faculty of Science, Cairo University, Giza 12613, Egypt; ^3^Department of Mathematics, Faculty of Science, Fayoum University, Fayoum 63514, Egypt

## Abstract

Two numerical algorithms based on dual-Petrov-Galerkin method are developed for solving the integrated forms of high odd-order boundary value problems (BVPs) governed by homogeneous and nonhomogeneous boundary conditions. Two different choices of trial functions and test functions which satisfy the underlying boundary conditions of the differential equations and the dual boundary conditions are used for this purpose. These choices lead to linear systems with specially structured matrices that can be efficiently inverted, hence greatly reducing the cost. The various matrix systems resulting from these discretizations are carefully investigated, especially their complexities and their condition numbers. Numerical results are given to illustrate the efficiency of the proposed algorithms, and some comparisons with some other methods are made.

## 1. Introduction

The spectral methods aim to approximate functions (solutions of differential equations) by means of truncated series of orthogonal polynomials. The main feature of spectral methods is to take various orthogonal systems of infinitely differentiable global functions as trial functions. The choice of different trial functions leads to different spectral approximations; for instance, the choice of trigonometric polynomials is suitable for periodic problems, while the choice of Chebyshev, Legendre, ultraspherical and classical Jacobi polynomials is suitable for nonperiodic problems. (see [1–7]).

Because of being extremely accurate, spectral methods have been intensively studied and successfully applied to numerical simulations in many fields. They have gained new popularity in automatic computations for a wide class of physical problems in fluid and heat flow. Mainly, there are three types of spectral methods, namely, collocation, tau, and Galerkin. The choice of the type of the method depends essentially on the application. Collocation methods are appropriate for studying nonlinear problems or when the problem has complicated coefficients, while Galerkin methods have the advantage of a more convenient analysis and optimal error analysis estimates. The tau method is applicable in the case of complicated (even nonlinear) boundary conditions, where Galerkin approach would be impossible and the collocation is extremely tedious (see [2, 5, 8]).

For the spectral solutions of odd-order differential equations by direct collocation methods, we obtain condition number of *O*(*N* ^2*k*^), where *N* is the number of retained modes and *k* is the order of equation. This high condition number will lead to instabilities caused by rounding errors (see [9, 10]).

High even-order boundary value problems have been investigated by a large number of authors because of both their mathematical importance and their potential for applications in hydrodynamic and hydromagnetic stability. Therefore, availability of fast and accurate algorithms to solve these equations will allow rapid solutions of many practical problems. Doha and Abd-Elhameed [11] and Doha et al. [12–14] have constructed efficient spectral-Galerkin algorithms using compact combinations of orthogonal polynomials for solving second and higher order equations.

Also, the study of odd-order equations is of interest; for example, the third-order equation is of fundamental mathematical interest since it lacks symmetry. Also, it is of physical interest since it contains a type of operator which appears in many commonly occurring partial differential equations such as the Korteweg-de Vries equation. Fifth-order boundary value problems arise in the mathematical modelling of viscoelastic flows (see [15, 16]). Some studies are concerned with third- and fifth-order differential equations in finite intervals (see [17, 18]). Doha and Abd-Elhameed [19] have constructed efficient spectral-Galerkin algorithms using compact combinations of ultraspherical polynomials for solving the differentiated forms of elliptic equations of (2*n* + 1)th-order. Recently, Bhrawy and Alghamdi [20], Doha et al. [21–23],

have analyzed some algorithms for solving numerically the third- and fifth-order differential equations.

The main objective of this paper is to develop some efficient spectral algorithms based on Legendre-dual-Petrov-Galerkin method (LDPGM) for the solution of the integrated forms of high odd-order BVPs in one variable. We present two different choices of appropriate bases for the LDPGM applied to high odd-order BVPs with homogenous and nonhomogenous boundary conditions. All the matrix systems resulted from the application of LDPGM to the integrated forms of (2*n* + 1)th-order differential equations are band, and then they are cheaper to solve than the systems obtained from the differentiated forms (see Doha and Abd-Elhameed [19]). This motivates our interest for applying LDPGM to the integrated forms of (2*n* + 1)th-order differential equations.

The remainder of the article is organized as follows. In Section 2, some properties of Legendre polynomials and their shifted ones are given. The main results of this paper are presented in Section 3, in which two numerical algorithms for solving the integrated forms of (2*n* + 1)th-order elliptic linear differential equations subject to homogeneous boundary conditions using two choices of bases functions are presented and implemented. Also, the same equations but governed by nonhomogeneous boundary conditions are noted in Section 3. For the sake of demonstrating the efficiency and the applicability of our two presented algorithms and also for the sake of comparison between these two algorithms, some numerical results are presented in Section 4. Some concluding remarks are given in Section 5.

## 2. Some Properties of Legendre Polynomials

The Legendre polynomials {*L*_*n*_(*x*), *n* = 0,1, 2,…} are a sequence of orthogonal polynomials on the interval (−1,1); that is,

(1) $$\begin{array}{c} \overset{1}{\underset{-1}{\int }}L_{k}\left(x\right)L_{j}\left(x\right)dx=\{\begin{array}{cc} 0, & k\neq j,\\ \frac{2}{2k+1}, & k=j. \end{array} \end{array}$$

The following special values of *L*_*k*_(*x*) and *D* ^*q*^ *L*_*k*_(*x*) = (*d* ^*q*^ *L*_*k*_(*x*)/*dx* ^*q*^) are important in our subsequent work:

(2) $$\begin{array}{c} L_{k}\left(-x\right)={\left(-1\right)}^{n}\;L_{k}\left(x\right),\;L_{k}\left(\pm 1\right)={\left(\pm 1\right)}^{k},\\ D^{q}L_{k}\left(\pm 1\right)=\frac{1}{2}\;{\left(\pm 1\right)}^{k+q}\overset{q-1}{\underset{m=0}{\prod }}\frac{\left(k-m\right)\left(k+m+1\right)}{\left(m+1\right)},\;\\ \;\;\;\;\;\;\;\;\;\;\;\;\;\;\;q=1,2,\ldots . \end{array}$$

The following two theorems and two lemmas are also needed hereafter.

**Theorem 1**

If the *q* times repeated integration of *L*_*k*_(*x*) is denoted by

(3) $$\begin{array}{c} I_{k}^{\left(q\right)}\left(x\right)=\overset{q\;times}{\overset{︷}{\int \int \cdots \int }}L_{k}\left(x\right)dx\;dx\cdots dx, \end{array}$$

then *I*_*k*_^(*q*)^(*x*) is given by

(4) $$\begin{array}{c} I_{k}^{\left(q\right)}\left(x\right)=\overset{q}{\underset{j=0}{\sum }}r_{j,k,q}L_{k+q-2j}\left(x\right)+\pi_{q-1}\left(x\right), \end{array}$$

where

(5) $$\begin{array}{c} r_{j,k,q}=\frac{{\left(-1\right)}^{j}\left(\begin{array}{c} q\\ j \end{array}\right)\left(k+q-2j+\left(1/2\right)\right)\Gamma \left(k-j+\left(1/2\right)\right)}{2^{q}\Gamma \left(k+q-j+\left(3/2\right)\right)}, \end{array}$$

and *π*_*q*−1_(*x*) is a polynomial of degree at most (*q* − 1).

(For the proof of Theorem 1, see Doha [3].)

**Theorem 2**

For all *j*, *m* ∈ *ℤ* ^+^, one has

(6) $$\begin{array}{c} x^{m}L_{j}\left(x\right)=\overset{m}{\underset{n=0}{\sum }}\lambda_{m,2n}\left(j\right)L_{j+m-2n}\left(x\right), \end{array}$$

where

(7) λm,2n(j)=2j+m−2n+1j!m!(j+m−2n+(1/2))  (j+m−2n)! ×∑k=max⁡(0,j−2n)min⁡(j+m−2n,j)(((j+m−2nk)(j+k)!)×(2k(2n+k−j)!×(3j+2m−4n−k+1)!)−1) ×∑ℓ=0j−k(((−1)ℓ(2j+m−2n−k−ℓ)!×(j+m−2n+ℓ)!)×(ℓ!(j−k−ℓ)!(j−ℓ)!(k+ℓ)!)−1) ×F12(−(k−j+2n),j+m−2n+ℓ+1;    3j+2m−4n−k+2;2).

(For the proof of Theorem 2, see, Doha [24].)

**Lemma 3**

For all *k*, *m* ∈ *ℤ* ^+^, one has

(8) $$\begin{array}{c} {\left(1-x^{2}\right)}^{m}L_{k}\left(x\right)=\overset{2m}{\underset{s=0}{\sum }}h_{s,m}\left(k\right)L_{k-2s+2m}\left(x\right), \end{array}$$

where

(9) $$\begin{array}{c} h_{s,m}\left(k\right)=\overset{m}{\underset{i=0}{\sum }}{\left(-1\right)}^{i}\left(\begin{array}{c} m\\ i \end{array}\right)\lambda_{2i,s+i-m}\left(k\right), \end{array}$$

and *λ*_*m*,*s*_(*k*) is defined as in relation (6).

**Proof**

By binomial theorem, we have

(10) $$\begin{array}{c} {\left(1-x^{2}\right)}^{m}L_{k}\left(x\right)=\overset{m}{\underset{s=0}{\sum }}{\left(-1\right)}^{s}\left(\begin{array}{c} m\\ s \end{array}\right)x^{2s}\;L_{k}\left(x\right); \end{array}$$

then, by using relation (6), we get

(11) (1−x2)mLk(x)=∑s=0m  ∑r=02s(−1)s(ms)λ2s,r(k)  Lk+2s−2r(x).

If we expand relation (11) and collect similar terms, then, after some rather algebraic manipulation, relation (8) is obtained. This completes the proof of Lemma 3.

**Lemma 4**

For all *k*, *m* ∈ *ℤ* ^+^, one has

(12) $$\begin{array}{c} {\left(1-x^{2}\right)}^{m}\left(1+x\right)L_{k}\left(x\right)=\overset{4m+2}{\underset{s=0}{\sum }}\;w_{s,m}\left(k\right)L_{k+2m-s+1}\left(x\right),\\ {\left(1-x^{2}\right)}^{m}\left(1-x\right)L_{k}\left(x\right)=\overset{4m+2}{\underset{s=0}{\sum }}\;{\overset{-}{w}}_{s,m}\left(k\right)L_{k+2m-s+1}\left(x\right), \end{array}$$

where

(13) ws,m(k) ={(k+2m+1)(2k+4m+1)h0,m(k),s=0,(k−2m+1)(2k−4m+1)h2m,m(k),s=4m+2,h(s−1)/2,m(k),s  odd,(k+2m−s+3)(2k+4m−s+5)h(s−2)/2,m(k) +(k+2m−s+1)(2k+4m−s+1)hs/2,m(k),s  even,

${\overset{-}{w}}_{s,m}(k)={\left(-1\right)}^{s+1}w_{s,m}(k)$ and *h*_*s*,*m*_(*k*) is defined as in Lemma 3.

**Proof**

This lemma can be immediately proved by multiplying both sides of relation (8) by (1 + *x*) and (1 − *x*), respectively, and performing some simple algebraic manipulations.

### 2.1. Shifted Legendre Polynomials

The shifted Legendre polynomials are defined on [*a*, *b*] as

(14) $$\begin{array}{c} L_{k}^{*}\left(x\right)=L_{k}\left(\frac{2x-a-b}{b-a}\right). \end{array}$$

All results of Legendre polynomials can be easily transformed to give the corresponding results for their shifted ones.

The orthogonality relation of *L*_k_*(*x*) on [*a*, *b*] is given by

(15) $$\begin{array}{c} \overset{b}{\underset{a}{\int }}L_{k}^{*}\left(x\right)L_{j}^{*}\left(x\right)dx=\{\begin{array}{cc} 0, & k\neq j,\\ \frac{b-a}{2k+1}, & k=j. \end{array} \end{array}$$

Now, based on relation (14) and with the aid of formula (4), we have the following theorem.

**Theorem 5**

If the *q* times repeated integration of *L*_k_*(*x*) is denoted by

(16) $$\begin{array}{c} J_{k}^{\left(q\right)}\left(x\right)=\overset{q\;times}{\overset{︷}{\int \int \cdots \int }}L_{k}^{*}\left(x\right)\overset{q\;times}{\overset{︷}{dx\;dx\;\cdots dx}}, \end{array}$$

then

(17) $$\begin{array}{c} J_{k}^{\left(q\right)}\left(x\right)={\left(\frac{b-a}{2}\right)}^{q}\overset{q}{\underset{j=0}{\sum }}r_{j,k,q}L_{k+q-2j}^{*}\left(x\right)+{\overset{-}{\pi }}_{q-1}\left(x\right), \end{array}$$

where *r*_*j*,*k*,*q*_ is defined as in (5) and ${\overset{-}{\pi }}_{q-1}(x)$ is a polynomial in *x* of degree (*q* − 1) at most.

**Remark 6**

For all *x* ∈ [*a*, *b*] and *k*, *m* ∈ *ℤ* ^+^, one has

(18) (x−a)m+1(b−x)mLk∗(x) =(b−a2)2m+1  ∑s=04m+2ws,m(k)Lk+2m−s+1∗(x),

(19) (x−a)m(b−x)m+1Lk∗(x) =(b−a2)2m+1  ∑s=04m+2w−s,m(k)Lk+2m−s+1∗(x),

where *w*_*s*,*m*_(*k*) and ${\overset{-}{w}}_{s,m}(k)$ are defined as in Lemma 4.

## 3. Solution of High Odd-Order BVPs

We are interested in using the LDPGM to solve the two-point high odd-order BVPs

(20) $$\begin{array}{c} u^{\left(2n+1\right)}\left(x\right)+\overset{2n}{\underset{i=0}{\sum }}\eta_{i}u^{\left(i\right)}\left(x\right)=f\left(x\right),\;x\in \left(a,b\right),\;\;n\geq 1, \end{array}$$

governed by the homogeneous boundary conditions

(21) $$\begin{array}{c} u^{\left(j\right)}\left(a\right)=u^{\left(j\right)}\left(b\right)=u^{\left(n\right)}\left(a\right)=0,\;j=0,1,\ldots ,n-1, \end{array}$$

where *u* ^(*j*)^(*x*) = *D* ^*j*^ *u*(*x*) denotes the *j*th derivative of *u*(*x*) with respect to *x* and {*η*_*i*_, *i* = 0,1,…, 2*n*} are known constant coefficients.

In this section, we consider two kinds of bases to numerically solve (20) governed by (21) but by considering its integrated form; namely,

(22) u(x)+∑i=02nηi∫(2n−i+1)u(x)(dx)(2n−i+1) =p(x)+∑i=02nbiLi∗(x),   x∈(a,b),u(j)(a)=u(j)(b)=u(n)(a)=0, j=0,1,…,n−1,

where

(23) $$\begin{array}{c} \overset{\left(q\right)}{\underset{}{\int }}u\left(x\right){\left(dx\right)}^{q}=\overset{q\;times}{\overset{︷}{\int \int \cdots \int }}u\left(x\right)\overset{q\;times}{\overset{︷}{dx\;dx\cdots dx}},\\ p\left(x\right)=\overset{\left(2n+1\right)}{\underset{}{\int }}f\left(x\right){\left(dx\right)}^{\left(2n+1\right)}, \end{array}$$

and {*b*_*i*_, *i* = 0,1,…, 2*n*} are the unknown constants of integration.

We set

(24) SN=span⁡{L0∗(x),L1∗(x),…,LN∗(x)},YN={y∈SN:y(j)(a)=y(j)(b)=y(n)(a)=0,    j=0,1,…,n−1},Y−N={y∈SN:y(j)(a)=y(j)(b)=y(n)(b)=0,    j=0,1,…,n−1},

and then the LDPGM for solving (20)-(21) or its equivalent integrated form (22) is to find *u*_*N*_^*n*^ ∈ *Y*_*N*_ such that

(25) (uNn(x),y(x))  +∑i=02nηi(∫(2n−i+1)uNn(x)(dx)(2n−i+1),y(x)) =(p(x)+∑i=02nbiLi∗(x),y(x)),      ∀y∈Y−N,

where (*u*, *v*) = ∫_*a*_^*b*^ *u*(*x*)*v*(*x*)*dx* is the scalar inner product in the space *L* ^2^(*a*, *b*). Chebyshev, Legendre,

In the following two subsections, we will apply the dual Petrov-Galerkin method to solve (22) based on choosing two kinds of bases functions. These two choices enable one to obtain two linear systems of specially structured matrices that can be efficiently inverted.

### 3.1. The First Choice of Basis Functions

First, we consider the case [*a*, *b*] = [−1,1] and set

(26) θk,n(x)=Lk(x)+∑m=12n+1dm,kLk+m(x), k=0,1,2,…,N−2n−1, n≥1,θ¯k,n(x)=Lk(x)+∑m=12n+1d−m,kLk+m(x), k=0,1,2,…,N−2n−1, n≥1.

We choose the coefficients {*d*_*m*,*k*_} and $\left\{{\overset{-}{d}}_{m,k}\right\}$ such that *θ*_*k*,*n*_(*x*) ∈ *Y*_*k*+2*n*+1_ and ${\bar{\theta }}_{k,n}(x)\in {\overset{-}{Y}}_{k+2n+1}$, respectively. The boundary conditions *θ*_*k*,*n*_^(*j*)^(*a*) = *θ*_*k*,*n*_^(*j*)^(*b*) = *θ*_*k*,*n*_^(*n*)^(*a*) = 0, *j* = 0,1,…, *n* − 1 lead to the following system of equations:

(27) ∑m=12n+1(−1)mdm,k=−1,∑m=12n+1(−1)m[∏s=0q−1(k+m−s)(k+m+s+1)] ×dm,k=−∏s=0q−1(k−s)(k+s+1),     q=1,2,…,n, n≥1,∑m=12n+1dm,k=−1,∑m=12n+1∏s=0q−1(k+m−s)(k+m+s+1)dm,k =−∏s=0q−1(k−s)(k+s+1),q=1,2,…,n−1, n≥1.

The determinant of the above system of linear equations is different from zero; hence, {*d*_*m*,*k*_} can be uniquely determined to give

(28)  dm,k=(−1)m{((−1)m/2(nm2)(k+m+12) ×Γ(k+m+12)Γ(k+n+32)) ×(Γ(k+32)Γ(k+n+m+32))−1,m  even,((−1)(m+1)/2(nm−12)(k+m+12) ×Γ(k+m2+1)Γ(k+n+32)) ×(Γ(k+32)Γ(k+n+m2+2))−1,m  odd.

Similarly, it can be easily shown that $\left\{{\overset{-}{d}}_{m,k}\right\}$ are given by

(29) $$\begin{array}{c} {\overset{-}{d}}_{m,k}={\left(-1\right)}^{m}\;d_{m,k},\;m=1,2,\ldots ,2n+1. \end{array}$$

Second, if we replace *x* in (26) by ((2*x* − *a* − *b*)/(*b* − *a*)), for *a* ≤ *x* ≤ *b*, and if we define

(30) $$\begin{array}{c} \theta_{k,n}\left(\frac{2x-a-b}{b-a}\right)=\phi_{k,n}\left(x\right),\\ \;\;\;\;{\overset{-}{\theta }}_{k,n}\left(\frac{2x-a-b}{b-a}\right)=\psi_{k,n}\left(x\right),\;x\in \left[a,b\right], \end{array}$$

then it is obvious that the basis functions and their dual are given, respectively, by

(31) $$\begin{array}{c} \phi_{k,n}\left(x\right)=\overset{2n+1}{\underset{m=0}{\sum }}d_{m,k}L_{k+m}^{*}\left(x\right), \end{array}$$

(32) $$\begin{array}{c} \psi_{k,n}\left(x\right)=\overset{2n+1}{\underset{m=0}{\sum }}{\overset{-}{d}}_{m,k}L_{k+m}^{*}\left(x\right), \end{array}$$

where *d*_*m*,*k*_ and ${\overset{-}{d}}_{m,k}$ are as given in (28) and (29), respectively.

Now it is clear that (25) is equivalent to

(33) (uNn(x),ψk,n(x))  +∑i=02nηi(∫(2n−i+1)uNn(x)(dx)(2n−i+1),ψk,n(x)) =(p(x)+∑i=02nbiLi∗(x),ψk,n(x)).

If we take *k* ≥ 2*n* + 1 in (33), then the constants *b*_*i*_, 0 ≤ *i* ≤ 2*n*, would disappear, and then we get

(34) (uNn(x),ψk,n(x))  +∑i=02nηi(∫(2n−i+1)uNn(x)(dx)(2n−i+1),ψk,n(x)) =(p(x),ψk,n(x)), 2n+1≤k≤N.

If we denote

(35) $$\begin{array}{c} p_{k}^{n}=\left(p\left(x\right),\psi_{k,n}\left(x\right)\right),\\ p^{n}={\left(p_{2n+1}^{n},p_{2n+2}^{n},\ldots ,p_{N}^{n}\right)}^{T},\\ u_{N}^{n}\left(x\right)=\overset{N-2n-1}{\underset{m=0}{\sum }}v_{m}^{n}\phi_{m,n}\left(x\right),\\ v^{n}={\left(v_{0}^{n},v_{1}^{n},\ldots ,v_{N-2n-1}^{n}\right)}^{T},\\ A_{n}={\left(a_{kj}^{n}\right)}_{2n+1\leq k,j\leq N},\\ \;\;\;\;B_{2n-i+1,n}={\left(b_{kj}^{2n-i+1,n}\right)}_{2n+1\leq k,j\leq N},\;0\leq i\leq 2n, \end{array}$$

then (34) is equivalent to the following matrix system:

(36) $$\begin{array}{c} \left(A_{n}+\overset{2n}{\underset{i=0}{\sum }}\eta_{i}B_{2n-i+1,n}\right)v^{n}=p^{n}, \end{array}$$

where the nonzero elements of the matrices *A*_*n*_ and *B*_2*n*−*i*+1,*n*_ (0 ≤ *i* ≤ 2*n*) are given explicitly in the following theorem.

**Theorem 7**

If the basis functions *ϕ*_*k*,*n*_(*x*) and their dual *ψ*_*k*,*n*_(*x*) are taken as in (31) and (32), respectively, and if we denote   *a*_*kj*_^*n*^ = (*ϕ*_*j*−2*n*−1,*n*_(*x*), *ψ*_*k*,*n*_(*x*)) and *b*_*kj*_^2*n*−*i*+1,*n*^ = (∫^(2*n*−*i*+1)^ *ϕ*_*j*−2*n*−1,*n*_(*x*)(*dx*)^(2*n*−*i*+1)^, *ψ*_*k*,*n*_(*x*)), 0 ≤ *i* ≤ 2*n*, then

(37) $$\begin{array}{c} Y_{N+2n+1}=span\left\{\phi_{0,n}\left(x\right),\phi_{1,n}\left(x\right),\ldots ,\phi_{N,n}\left(x\right)\right\},\\ {\overset{-}{Y}}_{N+2n+1}=span\left\{\psi_{0,n}\left(x\right),\psi_{1,n}\left(x\right),\ldots ,\psi_{N,n}\left(x\right)\right\}, \end{array}$$

and the nonzero elements of the matrices *A*_*n*_, *B*_2*n*−*i*+1,*n*_  (0 ≤ *i* ≤ 2*n*) are given explicitly by

(38) $$\begin{array}{c} a_{kk}^{n}=\frac{{\left(-1\right)}^{n+1}\left(b-a\right)\Gamma^{2}\left(k-n+\left(1/2\right)\right)}{2\Gamma \left(k+\left(3/2\right)\right)\Gamma \left(k-2n+\left(1/2\right)\right)}, \end{array}$$

(39) akjn=(−1)j−k−1(b−a) ×∑m=02n+1(−1)mdm,j−2n−1dj−k+m−2n−1,k(2j+2m−4n−1),         1≤j−k≤4n+2,

(40) bkj2n−i+1,n=(−1)j−k−i(b−a)2n−i+222n−i+1 ×∑m=02n+1  ∑ℓ=02n−i+1(((−1)m  dm, j−2n−1dj+m−i−2ℓ−k, k×rℓ, j−2n+m−1, 2n−i+1)×(2j+2m−2i−4ℓ+1)−1),i−2n−1≤j−k≤6n−i+3,

where *r*_*ℓ*,*j*,*q*_ and *d*_*m*,*j*_ are, respectively, defined as in (5) and (28).

**Proof**

We choose the basis functions *ϕ*_*k*,*n*_(*x*) and their dual *ψ*_*k*,*n*_(*x*) such that *ϕ*_*k*,*n*_(*x*) ∈ *Y*_*N*+2*n*+1_ and $\psi_{k,n}(x)\in {\overset{-}{Y}}_{N+2n+1}$ for *k* = 0,1,…, *N*. Moreover, it is clear that {*ϕ*_*k*,*n*_(*x*)} and {*ψ*_*k*,*n*_(*x*)} are linearly independent and the dimension of each of *Y*_*N*+2*n*+1_ and ${\overset{-}{Y}}_{N+2n+1}$ is equal to (*N* + 1). Hence,

(41) $$\begin{array}{c} Y_{N+2n+1}=\mathrm{span}\left\{\phi_{0,n}\left(x\right),\phi_{1,n}\left(x\right),\ldots ,\phi_{N,n}\left(x\right)\right\},\\ {\overset{-}{Y}}_{N+2n+1}=\mathrm{span}\left\{\psi_{0,n}\left(x\right),\psi_{1,n}\left(x\right),\ldots ,\psi_{N,n}\left(x\right)\right\}. \end{array}$$

The nonzero elements (*a*_*kj*_^*n*^) for 2*n* + 1 ≤ *k*, *j* ≤ *N*, can be obtained by making use of formulae (31) and (32). Now, *a*_*kj*_^*n*^ is given by

(42) akjn=(∑m=02n+1dm,j−2n−1Lj−2n+m−1∗(x),∑p=02n+1(−1)pdp,kLk+p∗(x))=∑m=02n+1∑p=02n+1(−1)pdm,j−2n−1dp,k(Lj−2n+m−1∗(x),Lk+p∗(x)),

which in turn, with the aid of the orthogonality relation (15), yields

(43) akjn=(−1)j−k−1(b−a)   ×∑m=02n+1(−1)mdm,j−2n−1dj−k+m−2n−1,k(2j+2m−4n−1),

which proves relation (39). From (43) and (28), it is not difficult to prove that

(44) $$\begin{array}{c} a_{kk}^{n}=\frac{{\left(-1\right)}^{n+1}\left(b-a\right)\Gamma^{2}\left(k-n+\left(1/2\right)\right)}{2\Gamma \left(k+\left(3/2\right)\right)\Gamma \left(k-2n+\left(1/2\right)\right)}. \end{array}$$

To prove relation (40), we have, for 0 ≤ *i* ≤ 2*n*,

(45) bkj2n−i+1,n=(∫(2n−i+1)ϕj−2n−1,n(x)(dx)(2n−i+1),ψk,n(x))=(∑m=02n+1dm,j−2n−1Jj−2n+m−1(2n−i+1)(x),∑p=02n+1(−1)p  dp,kLk+p∗(x)).

Making use of Theorem 5 enables one to write

(46) Jj−2n+m−1(2n−i+1)(x)=(b−a2)(2n−i+1) ×∑ℓ=02n−i+1rℓ,j−2n+m−1,2n−i+1    ×Lj+m−i−2ℓ∗(x)+π−2n−i(x),

where *r*_*j*,*k*,*q*_ is defined as in (5) and ${\overset{-}{\pi }}_{2n-i}$ is a polynomial in *x* of degree (2*n* − *i*) at most.

This, with the orthogonality relation (15), yields

(47) bkj2n−i+1,n=(−1)j−k−i(b−a)2n−i+222n−i+1 ×∑m=02n+1  ∑ℓ=02n−i+1(((−1)mdm,j−2n−1  dj+m−i−2ℓ−k,k‍ ×rℓ,j−2n+m−1,2n−i+1)×(2j+2m−2i−4ℓ+1)−1),

which completes the proof of Theorem 7.

### 3.2. The Second Choice of Basis Functions

As a second choice of *ϕ*_*k*,*n*_(*x*) and *ψ*_*k*,*n*_(*x*), we write

(48) $$\begin{array}{c} \phi_{k,n}\left(x\right)={\left(\frac{2}{b-a}\right)}^{2n+1}{\left(x-a\right)}^{n+1}{\left(b-x\right)}^{n}L_{k}^{*}\left(x\right), \end{array}$$

(49) $$\begin{array}{c} \psi_{k,n}\left(x\right)={\left(\frac{2}{b-a}\right)}^{2n+1}{\left(x-a\right)}^{n}{\left(b-x\right)}^{n+1}L_{k}^{*}\left(x\right), \end{array}$$

which automatically fulfill the boundary conditions (21) and their dual conditions, respectively.

The following lemma is of fundamental importance in what follows.

**Lemma 8**

For arbitrary constants *v*_*k*_^*n*^, one has

(50) $$\begin{array}{c} \overset{N-2n-1}{\underset{k=0}{\sum }}v_{k}^{n}\;\phi_{k,n}\left(x\right)=\overset{N}{\underset{k=0}{\sum }}e_{k}L_{k}^{*}\left(x\right), \end{array}$$

where

(51) $$\begin{array}{c} e_{k}=\overset{4n+2}{\underset{s=0}{\sum }}w_{s,n}\left(k-2n+s-1\right)v_{k-2n+s-1}^{n}. \end{array}$$

Moreover,

(52) ∫(q)(∑k=0N−2n−1  vkn  ϕk,n(x))(dx)(q) =∑k=0N+qtk,qLk∗(x)+ρq−1(x), 1≤q≤2n+1,

where

(53) tk,q=(b−a2)q ×∑j=0q∑s=04n+2ws,n(k−2n+s+2j−q−1)    ×rj,k−q+2j,qvk−2n+s+2j−q−1n,

and *w*_*s*,*n*_(*k*) is defined as in (13) and *ρ*_*q*−1_(*x*) is a polynomial of degree (*q* − 1) at most.

**Proof**

The first part of this lemma can be directly proved with the aid of Lemma 4. To prove the second part, we integrate both sides of (50) *q* times, 1 ≤ *q* ≤ 2*n* + 1, and, after making use of formula (17), we obtain

(54) ∫(q)(∑k=0N−2n−1vkn  ϕk,n(x))(dx)(q) =(b−a2)q∑k=0Nek∑j=0qrj,k,qLk+q−2j∗(x)+ρq−1,

which may be written in the form

(55) $$\begin{array}{c} \overset{\left(q\right)}{\underset{}{\int }}\left(\overset{N-2n-1}{\underset{k=0}{\sum }}v_{k}^{n}\;\phi_{k,n}\left(x\right)\right){\left(dx\right)}^{\left(q\right)}=\overset{N+q}{\underset{k=0}{\sum }}t_{k,q}L_{k}^{*}\left(x\right)+\rho_{q-1}, \end{array}$$

where

(56) $$\begin{array}{c} t_{k,q}={\left(\frac{b-a}{2}\right)}^{q}\overset{q}{\underset{j=0}{\sum }}e_{k-q+2j}r_{j,k-q+2j,q}. \end{array}$$

Now, making use of relation (51) enables one to write *t*_*k*,*q*_ in the form

(57) tk,q=(b−a2)q ×∑j=0q∑s=04n+2ws,n(k−2n+s+2j−q−1)    ×rj,k−q+2j,qvk−2n+s+2j−q−1n,

and this completes the proof of Lemma 8.

**Theorem 9**

If *u*_*N*_^*n*^(*x*) = ∑_*k*=0_^*N*−2*n*−1^ *v*_*k*_^*n*^ *ϕ*_*k*,*n*_(*x*) is the dual Petrov-Galerkin approximation to (20) and (21), then the expansion coefficients {*v*_*k*_^*n*^ : *k* = 0,1,…, *N* − 2*n* − 1} satisfy the matrix system

(58) $$\begin{array}{c} \left({\overset{-}{A}}_{n}+\overset{2n+1}{\underset{q=1}{\sum }}\eta_{2n-q+1}{\overset{-}{B}}_{q,n}\right)v^{n}=p^{*n}, \end{array}$$

where the nonzero elements of the matrices ${\overset{-}{A}}_{n}$ and ${\overset{-}{B}}_{q,n},\;1\leq q\leq 2n+1$ are given by

(59) a−kkn=k!Γ(k−2n−(1/2))22n+1(k−2n−1)!Γ(k+(1/2)),

(60) a−kjn=wj−k,n(j−2n−1), k≤j≤k+4n+2,

(61) b−kjq,n=(b−a2)q∑s=0qwj−k+q−2s,n(j−2n−1)rs,k−q+2s,q,k−q≤j≤k+4n+q+2,

where *r*_*ℓ*,*j*,*q*_ and *w*_*s*,*n*_ are, respectively, defined as in (5) and (13).

**Proof**

If we apply the LDPGM to (20), then we have

(62) (uNn(x)+∑q=12n+1η2n+1−q∫(q)uNn(dx)(q),ψk,n(x)) =(p(x)+∑i=02nξiLi∗(x),ψk,n(x)),

where *u*_*N*_^*n*^(*x*) = ∑_*k*=0_^*N*−2*n*−1^ *v*_*k*_^*n*^ *ϕ*_*k*,*n*_(*x*),  *ϕ*_*k*,*n*_(*x*) and *ψ*_*k*,*n*_(*x*) are defined as in (48) and (49), respectively.

Substitution of formulae (19), (50), and (52) into (62) yields

(63) (∑s=0NesLs∗(x)+∑q=12n+1η2n−q+1∑s=0N+qts,qLs∗(x),    ∑ℓ=04n+2w−ℓ,n(k)Lk−ℓ+2n+1∗(x)) =(p(x)+∑i=02nξiLi∗(x),∑ℓ=04n+2w−ℓ,n(k)Lk−ℓ+2n+1∗(x)).

The constants of integration *ξ*_*i*_, 0 ≤ *i* ≤ 2*n* would disappear if we take *k* ≥ 2*n* + 1, and therefore the application of the orthogonality relation (15) yields

(64) $$\begin{array}{c} \left(\frac{b-a}{2k+1}\right)\left(e_{k}+\overset{2n+1}{\underset{q=1}{\sum }}\eta_{2n+1-q}t_{k,q}\right)=p_{k}^{n},\\ \;\;\;\;\;\;\;k=2n+1,2n+2,\ldots ,N, \end{array}$$

which is equivalent to

(65) $$\begin{array}{c} e_{k}+\overset{2n+1}{\underset{q=1}{\sum }}\eta_{2n+1-q}t_{k,q}=p_{k}^{*n}\;k=2n+1,2n+2,\ldots ,N, \end{array}$$

(66) $$\begin{array}{c} p_{k}^{*n}=\frac{\left(2k+1\right)p_{k}^{n}}{\left(b-a\right)}. \end{array}$$

The system of linear equation (65) in turn may be written in the following matrix form:

(67) $$\begin{array}{c} \left({\overset{-}{A}}_{n}+\overset{2n+1}{\underset{q=1}{\sum }}\eta_{2n-q+1}{\overset{-}{B}}_{q,n}\right)v^{n}=p^{*n}, \end{array}$$

and this completes the proof of Theorem 9.

### 3.3. Structure and Complexities of the Two Matrix Systems (36) and (58)

The structures of the coefficient matrices $A_{n},B_{2n-i+1,n}\;\;(0\leq i\leq 2n),{\overset{-}{A}}_{n}$, ${\overset{-}{B}}_{q,n}\;\;(1\leq q\leq 2n)$ and hence the structures of the two combined matrices *D*_*n*_ = *A*_*n*_ + ∑_*i*=0_^2*n*^ *η*_*i*_ *B*_2*n*−*i*+1,*n*_ and ${\overset{-}{D}}_{n}={\overset{-}{A}}_{n}+\sum_{q=1}^{2n+1}\eta_{2n-q+1}{\overset{-}{B}}_{q,n}$, which appear in the two linear systems (36) and (58), will be discussed in this section. Also, the influence of such structures on the efficiency of the solution of these two matrix systems will be discussed.

With respect to the two matrices *A*_*n*_ and ${\overset{-}{A}}_{n}$, they are always nonsingular special upper band triangular of bandwidth (4*n* + 2), ∀*n* ≥ 1. Therefore, it is worth noting here that the case whichcorresponds to *η*_*i*_ = 0, 0 ≤ *i* ≤ 2*n*, leads to linear systems with special nonsingular upper triangular matrices. The results for such case are summarized in the following two important corollaries.

**Corollary 10**

If *η*_*i*_ = 0, 0 ≤ *i* ≤ 2*n*, then the system (36) takes the form *A*_*n*_ **v** ^*n*^ = **p** ^*n*^, where *A*_*n*_ is an upper triangular matrix whose solution can be obtained directly by the backward substitution

(68) $$\begin{array}{c} v_{k}^{n}=\frac{p_{k+2n+1}^{n}-\sum_{j=k+1}^{k+4n+2}a_{k+2n+1,j+2n+1}^{n}v_{j}^{n}}{a_{k+2n+1,k+2n+1}^{n}},\\ \;\;\;\;\;\;\;\;k=0,1,\ldots ,N-2n-1, \end{array}$$

where *a*_*kk*_^*n*^ and *a*_*kj*_^*n*^ are given by (38) and (39), respectively.

**Corollary 11**

If *η*_2*n*−*q*+1_ = 0, 1 ≤ *q* ≤ 2*n* + 1, then the system (58) takes the form ${\overset{-}{A}}_{n}\;v^{n}=p^{*n}$,    where ${\overset{-}{A}}_{n}$ is an upper triangular matrix whose solution can be obtained directly by the backward substitution

(69) $$\begin{array}{c} v_{k}^{n}=\frac{p_{k+2n+1}^{*n}-\sum_{j=k+1}^{k+4n+2}{\overset{-}{a}}_{k+2n+1,\;\;j+2n+1}^{n}\;v_{j}^{n}}{{\overset{-}{a}}_{k+2n+1,\;\;k+2n+1}^{n}},\\ \;\;\;\;\;\;\;k=0,1,\ldots ,N-2n-1, \end{array}$$

where ${\overset{-}{a}}_{kk}^{n}$ and ${\overset{-}{a}}_{kj}^{n}$ are given by (59) and (60), respectively.

Each of the matrices *B*_*q*,*n*_ and ${\overset{-}{B}}_{q,n}\;\;(1\leq q\leq 2n+1)$ is a band matrix whose total number of nonzero diagonals upper the main diagonal is 4*n* + *q* + 2, while the total number of nonzero diagonals lower the main diagonal is *q*. Thus, the coefficient matrices *D*_*n*_ = *A*_*n*_ + ∑_*q*=1_^2*n*+1^ *η*_2*n*−*q*+1_ *B*_*q*,*n*_ and ${\overset{-}{D}}_{n}={\overset{-}{A}}_{n}+\sum_{q=1}^{2n+1}\eta_{2n-q+1}{\overset{-}{B}}_{q,n}$ are band whose total number of nonzero diagonals upper the main diagonal does not exceed 6*n* + 3 and the total number of nonzero diagonals lower the main diagonal does not exceed 2*n* + 1. These special structures of *D*_*n*_ and ${\overset{-}{D}}_{n}$ simplify greatly the solutions of the two linear systems (36) and (58). The two systems in such case can be factorized by *LU*-decomposition, and the number of operations necessary to construct this factorization is of order 12(*N* − 2*n* − 1)(2*n* + 1)^2^ (see Stewart [28]).

### 3.4. Condition Number

Whenever spectral methods are used for solving the (2*n* + 1)th-order equations, one should be concerned with round-off errors caused by potentially large condition numbers. However, the LDPGM presented in this paper leads to systems with small condition numbers. For LDPGM, the two linear systems which resulted from the integrated form of the equation (*u* ^(2*n*+1)^(*x*) = *f*(*x*)) using the two choices of basis functions are given, respectively, by *A*_*n*_ **v** ^*n*^ = **p** ^*n*^ and ${\overset{-}{A}}_{n}v^{n}=p^{*n}$, where the matrices *A*_*n*_ and ${\overset{-}{A}}_{n},n\geq 1$ are upper triangular matrices whose diagonal elements are given by (38) and (59), respectively. Thus, for all *n* ≥ 1, we note that the condition number of the matrix *A*_*n*_ behaves like *O*(*k*) for large values of *k*, while the condition number of the matrix *D*_*n*_ is independent of *k*. This means that the matrix *D*_*n*_ is well conditioned. Hence, the propagation of round-off errors should not be very significant.

Table 1 illustrates the condition numbers for the two matrices *A*_*n*_ and *D*_*n*_ = *A*_*n*_ + ∑_*q*=1_^2*n*+1^ *η*_2*n*−*q*+1_ *B*_*q*,*n*_ in (36) for some values of the parameter *N* and (*a*, *b*) = (−1,1), while Table 2 illustrates the condition numbers for the two matrices ${\overset{-}{A}}_{n}$ and ${\overset{-}{D}}_{n}={\overset{-}{A}}_{n}+\sum_{q=1}^{2n+1}\eta_{2n-q+1}{\overset{-}{B}}_{q,n}$ in (58) (1 ≤ *n* ≤ 3) for the same values of *N* and in the same interval.

### 3.5. Nonhomogeneous Boundary Conditions

If we consider the differential equation

(70) $$\begin{array}{c} u^{\left(2n+1\right)}\left(x\right)+\overset{2n}{\underset{i=0}{\sum }}\eta_{i}u^{\left(i\right)}\left(x\right)=f\left(x\right),\;x\in \left(a,b\right),\;\;n\geq 1, \end{array}$$

governed by the nonhomogeneous boundary conditions

(71) u(j)(a)=αj, u(j)(b)=βj, u(n)(a)=γ,j=0,1,…,n−1,

one can easily show that the differential equation (70) with its nonhomogeneous boundary conditions (71) can be transformed—by using a suitable transformation—to a differential equation governed by homogeneous boundary conditions, but details will not be given here and the interested reader is referred to Doha and Abd-Elhameed [19].

## 4. Numerical Results

In this section, we give some numerical results obtained by using the two algorithms presented in the previous sections.

**Example 1**

Consider the following third-order one-dimensional BVP:

(72) u′′′(x)+η2u′′(x)+η1u′(x)+η0u(x)=f(x),u(±1)=u′(−1)=0,

where *f*(*x*) is chosen such that the exact solution of (72) is *u*(*x*) = (1 − *x* ^2^)(1 + *x*)*e* ^*x*^.

Table 3 lists the maximum pointwise error *E* given by |*u* − *u*_*N*_| to the problem (72) of the given example using LDPGM with the two choices of basis functions for various values of *N* and for some values of the coefficients *η*_0_,  *η*_1_, and *η*_2_.

**Example 2**

Consider the boundary value problem (see Lang and Xu [25] and Siddiqi and Akram [26]):

(73) $$\begin{array}{c} u^{\left(5\right)}\left(x\right)-u\left(x\right)=-\left(15+10x\right)e^{x},\;0\leq x\leq 1,\\ u\left(0\right)=0,\;u^{\prime }\left(0\right)=1,\;u^{\prime \prime }\left(0\right)=0,\\ u\left(1\right)=0,\;u^{\prime }\left(1\right)=-e, \end{array}$$

with exact solution *u*(*x*) = *x*(1 − *x*)*e* ^*x*^.

Table 4 lists the maximum pointwise error *E* given by |*u* − *u*_*N*_| using LDPGM with the two choices of basis functions and for various values of *N*. In Table 5, we give a comparison between the best errors obtained by our two methods (LDPGM-1st choice and LDPGM-2nd choice), quartic spline method (QSM) in [25], and sextic spline method (SSM) in [26]. This table shows that our methods are more accurate if compared with quartic spline method and sextic spline method illustrated in [25, 26], respectively.

**Example 3**

Consider the linear ninth-order BVP (see Wazwaz [27]):

(74) $$\begin{array}{c} u^{\left(9\right)}\left(x\right)-u\left(x\right)=-9e^{x},\;0<x<1,\\ u^{\left(j\right)}\left(0\right)=\left(1-j\right),\;0\leq j\leq 4,\\ u^{\left(j\right)}\left(1\right)=-je,\;0\leq j\leq 3, \end{array}$$

with theoretical solution *u*(*x*) = (1 − *x*)*e* ^*x*^.

In Table 6, we list the errors *E* given by |*u* − *u*_*N*_| for Example 3 for *N* = 16 using LDPGM based on the two kinds of basis functions. For the sake of comparison with the results obtained by Wazwaz in [27], the best errors obtained in [27] are listed in column 4 of Table 6. This table shows that our two algorithms are more accurate than the method presented by Wazwaz [27], for the problem considered.

## 5. Concluding Remarks

We have presented two numerical spectral algorithms for the solution of high odd-order differential equations based on Legendre-dual-Petrov-Galerkin method. The two presented algorithms are very reliable and efficient. The main advantage of our algorithms is that all the resulting systems are band and this of course simplifies the numerical computational efforts required to solve them. The presented numerical results exhibit the high accuracy and efficiency of the proposed algorithms.

## Figures and Tables

**Table 1:** Condition numbers for the matrices *A*_*n*_, *D*_*n*_.

*N*	*n*	Cond (*A*_*n*_)	(Cond (A_n_))/N	Cond (*D*_*n*_)	(Cond (D_n_))/N
12	1	2.347	1.956 · 10^−1^	3.120	2.600 · 10^−1^
24		4.387	1.828 · 10^−1^	5.461	2.275 · 10^−1^
32		5.754	1.798 · 10^−1^	7.039	2.199 · 10^−1^
48		8.493	1.769 · 10^−1^	10.191	2.123 · 10^−1^
64		1.123	1.755 · 10^−1^	13.333	2.083 · 10^−1^
12	2	1.379	1.149 · 10^−1^	2.263	1.886 · 10^−1^
24		1.756	7.315 · 10^−2^	2.569	1.071 · 10^−1^
32		2.218	6.932 · 10^−2^	3.147	9.836 · 10^−2^
48		3.160	6.584 · 10^−2^	4.349	9.059 · 10^−2^
64		4.109	6.421 · 10^−2^	5.564	8.694 · 10^−2^
12	3	2.657	2.214 · 10^−1^	3.208	2.673 · 10^−1^
24		2.780	1.159 · 10^−1^	3.683	1.535 · 10^−1^
32		2.780	8.689 · 10^−2^	3.683	1.151 · 10^−1^
48		2.780	5.793 · 10^−2^	3.683	7.673 · 10^−2^
64		2.780	4.344 · 10^−2^	3.683	5.755 · 10^−2^

**Table 2:** Condition numbers for the matrices ${\overset{-}{A}}_{n},{\overset{-}{D}}_{n}$.

*N*	*n*	Cond (${\overset{-}{A}}_{n}$)	Cond (${\overset{-}{D}}_{n}$)	*n*	Cond (${\overset{-}{A}}_{n}$)	Cond (${\overset{-}{D}}_{n}$)	*n*	Cond (${\overset{-}{A}}_{n}$)	Cond (${\overset{-}{D}}_{n}$)
12	1	2.781	10.003	2	3.127	8.327	3	3.127	6.759
24		2.996	10.152		3.620	9.105		4.026	8.866
32		3.048	10.152		3.735	9.107		4.224	8.899
48		3.099	10.152		3.847	9.107		4.414	8.899
64		3.124	10.152		3.902	9.106		4.506	8.899

**Table 3:** Maximum pointwise error *E* for Example 1.

*N*	*η*_0_	*η*_1_	*η*_2_	LDPG—1st choice	LDPG—2nd choice
18	0	0	0	2.20843 × 10^−14^	5.40665 × 10^−15^
20				3.06209 × 10^−14^	6.58881 × 10^−15^
22				7.61484 × 10^−14^	3.63132 × 10^−15^
18	1	− 1	− 1	1.14394 × 10^−13^	5.25079 × 10^−15^
20				1.00142 × 10^−13^	2.18044 × 10^−15^
22				2.17211 × 10^−13^	5.40459 × 10^−15^
18	16	−16^2^	−16^3^	1.15044 × 10^−13^	2.02084 × 10^−15^
20				1.65338 × 10^−14^	1.08947 × 10^−14^
22				5.91763 × 10^−13^	7.53041 × 10^−14^

**Table 4:** Maximum pointwise error *E* for Example 2 with *N* = 14,16,18,20.

*N*	LDPG—1st choice	LDPG—2nd choice
14	2.66574 × 10^−15^	1.36447 × 10^−15^
16	4.18619 × 10^−16^	1.3787 × 10^−16^
18	9.4369 × 10^−16^	1.70355 × 10^−16^
20	1.28588 × 10^−15^	2.01205 × 10^−16^
24	5.1337 × 10^−16^	1.80926 × 10^−16^

**Table 5:** Comparison between different methods for Example 2.

Error	LDPG—1st choice	LDPG—2nd choice	QSM in [25]	SSM in [26]
*E*	4.18619 × 10^−16^	1.3787 × 10^−16^	1.2331 × 10^−10^	5.2812 × 10^−7^

**Table 6:** Error *E* for Example 3 with *N* = 16.

*x*	LDPG—1st choice	LDPG—2nd choice	Method in Wazwaz [27]
0.0	0.000000	0.000000	0.000000
0.1	2.22045 × 10^−16^	2.22045 × 10^−16^	2.0 × 10^−10^
0.2	1.11022 × 10^−16^	1.11022 × 10^−16^	2.0 × 10^−10^
0.3	1.11022 × 10^−16^	1.11022 × 10^−16^	2.0 × 10^−10^
0.4	1.11022 × 10^−16^	1.11022 × 10^−16^	2.0 × 10^−10^
0.5	1.11022 × 10^−16^	0.000000	2.0 × 10^−10^
0.6	1.11022 × 10^−16^	1.11022 × 10^−16^	6.0 × 10^−10^
0.7	1.11022 × 10^−16^	0.000000	1.0 × 10^−9^
0.8	0.000000	1.11022 × 10^−16^	2.0 × 10^−9^
0.9	0.000000	0.000000	3.4 × 10^−9^
0.1	2.22045 × 10^−16^	2.22045 × 10^−16^	0.000000
